# Possible Interactions of Extracellular Loop IVP2-S6 With Voltage-Sensing Domain III in Cardiac Sodium Channel

**DOI:** 10.3389/fphar.2021.742508

**Published:** 2021-10-14

**Authors:** Anastasia K. Zaytseva, Aleksandr S. Boitsov, Anna A. Kostareva, Boris S. Zhorov

**Affiliations:** ^1^ Almazov National Medical Research Centre, St. Petersburg, Russia; ^2^ Sechenov Institute of Evolutionary Physiology and Biochemistry, Russian Academy of Sciences, St. Petersburg, Russia; ^3^ Department of Womenʼs and Childrenʼs Health, Karolinska Institute, Stockholm, Sweden; ^4^ Department of Biochemistry and Biomedical Sciences, McMaster University, Hamilton, ON, Canada

**Keywords:** sodium channelopathies, Brugada syndrome, cardiac arrhythmias, fast inactivation, slow inactivation, channel gating

## Abstract

Motion transmission from voltage sensors to inactivation gates is an important problem in the general physiology of ion channels. In a cryo-EM structure of channel hNa_v_1.5, residues N1736 and R1739 in the extracellular loop IVP2-S6 approach glutamates E1225 and E1295, respectively, in the voltage-sensing domain III (VSD-III). ClinVar-reported variants E1230K, E1295K, and R1739W/Q and other variants in loops IVP2-S6, IIIS1-S2, and IIIS3-S4 are associated with cardiac arrhythmias, highlighting the interface between IVP2-S6 and VSD-III as a hot spot of disease mutations. Atomic mechanisms of the channel dysfunction caused by these mutations are unknown. Here, we generated mutants E1295R, R1739E, E1295R/R1739E, and N1736R, expressed them in HEK-293T cells, and explored biophysical properties. Mutation E1295R reduced steady-state fast inactivation and enhanced steady-state slow inactivation. In contrast, mutation R1739E slightly enhanced fast inactivation and attenuated slow inactivation. Characteristics of the double mutant E1295R/R1739E were rather similar to those of the wild-type channel. Mutation N1736R attenuated slow inactivation. Molecular modeling predicted salt bridging of R1739E with the outermost lysine in the activated voltage-sensing helix IIIS4. In contrast, the loss-of-function substitution E1295R repelled R1739, thus destabilizing the activated VSD-III in agreement with our data that E1295R caused a depolarizing shift of the G-V curve. *In silico* deactivation of VSD-III with constraint-maintained salt bridge E1295-R1739 resulted in the following changes: 1) contacts between IIIS4 and IVS5 were switched; 2) contacts of the linker-helix IIIS4-S5 with IVS5, IVS6, and fast inactivation tripeptide IFM were modified; 3) contacts of the IFM tripeptide with helices IVS5 and IVS6 were altered; 4) mobile loop IVP2-S6 shifted helix IVP2 that contributes to the slow inactivation gate and helix IVS6 that contributes to the fast inactivation gate. The likelihood of salt bridge E1295-R1739 in deactivated VSD-III is supported by Poisson–Boltzmann calculations and state-dependent energetics of loop IVP2-S6. Taken together, our results suggest that loop IVP2-S6 is involved in motion transmission from VSD-III to the inactivation gates.

## Introduction

Voltage-gated sodium channels play key roles in the physiology and pathophysiology of excitable cells (Ahern et al., 2016; Catterall, 2017; Schwartz et al., 2020). The entry of sodium ions into the cell generates the rising phase of the action potential. The human genome encodes nine sodium channel isoforms (Na_v_1.x). The pore-forming α-subunit folds from a single-polypeptide chain of four homologous repeat domains (I–IV). Each repeat has a voltage-sensing domain (VSD) of four transmembrane helices (S1–S4). Upon membrane depolarization, the voltage-sensing helices S4 with positively charged arginine or lysine residues move in the extracellular direction. Each repeat contributes to the pore domain (PD), the pore-lining inner helix (S6), the outer helix (S5), and the membrane re-entering extracellular loop (P) between S5 and S6 that contains the membrane-descending (P1) and membrane-ascending (P2) helices. Residues Asp, Glu, Lys, and Ala between the P1 and P2 helices in repeats I, II, III, and IV, respectively, contribute to the selectivity filter (DEKA ring). The ring of outer carboxylates (EEDD) has residues Glu, Glu, Asp, and Asp in repeats I, II, III, and IV, respectively. The selectivity filter divides the ion permeation pathway into two parts: the outer pore exposed to the extracellular space and the inner pore, which merges with the cytoplasm in the open PD.

Sodium channels exist in various open, closed, and inactivated states. In the resting cell, helices S4 are electrostatically attracted to the cytoplasmic side of the hyperpolarized membrane, and PD is closed. Upon membrane depolarization, helices S4 shift in the extracellular direction. The shifts are transmitted to helices S5 and S6 through linker helices S4-S5. The activation gate, which is formed by hydrophobic residues at the C-terminal halves S6s, opens and sodium ions flow into the cell. A few milliseconds after activation, the sodium channel transits to the fast inactivation state due to binding of the IFM tripeptide in the III/IV linker to the hydrophobic cleft between helices IIIS5, IIIS6, IVS5, and IVS6. The IFM tripeptide does not occlude the pore (Pan et al., 2018) but shifts helices IIIS6 and IVS6 so that the activation gate closes. Membrane depolarization during hundreds of milliseconds or multiple prolonged depolarizations cause conformational changes at the outer pore and the channels transit into the slow inactivation state(s), thus reducing the inward sodium current (Silva, 2014; Ghovanloo et al., 2016; Chatterjee et al., 2018).

Electromechanical coupling of the voltage sensors and the inactivation gates is an important problem in the physiology of ion channels (Horn, 2000; Blunck and Batulan, 2012; Fernandez-Marino et al., 2018; Bassetto et al., 2021; Cowgill and Chanda, 2021). In potassium channels, the coupling involves interactions of the linker helices S4-S5 with helices S6 (Lu et al., 2002) and/or interactions between helices S4 and S5 (Fernandez-Marino et al., 2018; Carvalho-de-Souza and Bezanill, 2019). A recent study has revealed a chain of residues in the extracellular parts of S4, S5, and P-loop, which mediates functional connectivity between the VSD and the slow inactivation gate at the selectivity filter (Bassetto et al., 2021), henceforth referred to as the SF gate.

Earlier, we identified genetic variant A1294G of the hNa_v_1.5 channel in a patient with a combined clinical phenotype and demonstrated that this mutation negatively shifted the steady-state inactivation, accelerated the fast and slow inactivation, and decelerated recovery from the intermediate inactivation (Zaytseva et al., 2019). Glycine substitution of A1294, which is located in the extracellular linker IIIS3-S4, would increase the linker flexibility and thus affect intersegment contacts involving the linker and flanking extracellular residues in helices S3 and S4. In all available cryo-EM structures of the Na_v_1.x channels, a semirigid 15-membered disulfide-fastened loop IVP2-S6 hangs over the voltage-sensing domain III (VSD-III). Many ClinVar-reported disease mutations of Na_v_1.5 are located at the interface between VSD-III and extracellular loop IVP2-S6, implying functional importance of the interface. In particular, ClinVar-reported variants E1230K and E1231K in linker IIIS1-S2, E1295K in linker IIIS3-S4 and R1739W/Q in loop IVP2-S6 are associated with Brugada syndrome (BrS).

In the cryo-EM structure of the hNa_v_1.5 channel (PDB ID: 6lqa), residues E1230 and E1295 in the VSD-III approach, respectively, N1736 and R1739 in loop IVP2-S6. Direct contacts between the residues are lacking, but molecular modeling shows that such contacts may be imposed with minimal deformations of the backbones. Here, we generated mutants E1295R, R1739E, E1295R/R1739E, and N1736R and explored their electrophysiological properties in HEK-293T cells. We further constructed molecular models of the four channel mutants and *in silico* deactivated VSD-III. Our experimental data and molecular models suggest that electrostatic interactions between VSD-III and loop IVP2-S6 may contribute to motion transmission from VSD-III to the SF gate at the N-end of helix IVP2 and the fast inactivation gate at the C-end of helix IVS6.

## Methods

### Molecular Biology

Vector pcDNA3.1 with WT hNa_v_1.5 and GFP (hH1-pcDNA3.1) was kindly provided by Prof. Hugues Abriel (Institute of Biochemistry and Molecular Medicine, University of Bern, Switzerland). Site-directed mutagenesis was performed by PCR amplification according to the standard mutagenesis protocol with overlapped primers. hH1-pcDNA3.1 (1 µg) or mutant-pcDNA3.1 (1 µg) were transfected into the HEK-293T cells growing on 3 cm plates using 1 mg/ml water solution of linear polyethylenimine hydrochloride (PEI, MW 40,000, Polysciences) at 2:1 v/w ratio with pDNA. The cells were maintained in the DMEM medium supplemented with 2 mM glutamine, 100 U/ml penicillin, and 100 µg/ml streptomycin (Thermo Fisher Scientific) in a CO_2_ incubator at +37°C for 24 h and then seeded at poly-lysine (Sigma Aldrich) coated glasses for electrophysiological recordings.

### Electrophysiology

Recordings of sodium current I_Na_ were performed using the patch-clamp technique in the whole-cell configuration at room temperature. The extracellular solution for the current recordings contained the following (mmol/L): 140 NaCl, 1 MgCl2, 1.8 CaCl2, 10 HEPES, and 10 Glucose (pH 7.4 CsOH). The intracellular solution contained (mmol/L): 130 CsCl, 10NaCl, 10 EGTA, 10 HEPES (pH 7.3 CsOH). Microelectrodes were made from the borosilicate glass using a puller (P-1000, Sutter Instrument). The electrode resistance was 1.8–2.5 MΩ. The series resistance was compensated at 75–80%. Data acquisition was performed using amplifier Axopatch 200B and software Clampfit version 10.3 (Molecular Devices). Currents were acquired at 20–50 kHz and low-pass filtered at 5 kHz using an analog-to-digital interface (Digidata 1440A Acquisition System, Molecular Devices). Experiments were performed using at least three independent transfections.

### Data Analysis

We used the holding potential of −100 mV. Current-voltage (I-V) curves were accessed by depolarizing voltage steps from −80 to 60 mV during 40 ms in 5 mV increments at 1 Hz frequency. The current densities at each test potential were measured by dividing I_Na_ by the cell capacitance. The maximal I_Na_ at each voltage was obtained and the corresponding conductance (G) was calculated using equation G = I_Na_/(V-V_rev_), where V is the voltage test. The normalized G values were plotted against the voltage, and the G-V curves, which characterize the steady-state activation, were fitted to the Boltzmann function G/G_max_ = 1/(1 + exp ((V_1/2_ -V)/k)), where G_max_ is the maximal sodium conductance, V_1/2_ is the potential of half-maximal activation, and k is the slope factor.

The voltage dependence of the steady-state inactivation was tested by measuring I_Na_ elicited by a 20 ms step to −15 mV after a prepulse of 500 ms ranging from −120 to 0 mV in 5 mV steps. The normalized I_Na_ was plotted against the prepulse voltage. The steady-state inactivation curves were fitted with the Boltzmann function. The voltage dependency of the steady-state fast inactivation was obtained as in the previous protocol, but with 20 ms prepulse. The steady-state slow inactivation data were examined with a 10 s prepulse followed by 20 ms hyperpolarization to -100 mV to allow recovery from the fast inactivation.

### Statistical Analysis

All data are expressed as the mean values and standard errors (SEM). Statistical comparisons were made using the unpaired Mann–Whitney test with *p* < 0.05 considered to be statistically significant. In some figures, the standard error bars are smaller than the data symbols.

### Molecular Modeling

The methodology of our molecular modeling approach with the ZMM program is described, e.g., in (Bruhova and Zhorov, 2010; Garden and Zhorov, 2010; Tikhonov and Zhorov, 2017). Nonbonded interactions were calculated with the AMBER force field (Weiner et al., 1986) with the distance cutoff of 9 Å and a shifting function (Brooks et al., 1985). Electrostatic interactions were calculated with the distance- and environment-dependent dielectric function (Garden and Zhorov, 2010). Electrostatic interactions involving ionized groups were calculated without any cutoff. We used the cryo-EM structure of the human Na_v_1.5 channel (Li et al., 2021) as a template to build models of the hNa_v_1.5 mutants. The models were optimized by the Monte Carlo energy minimizations (MCM) (Li and Scheraga, 1987) in the space of generalized coordinates (Zhorov, 1981), which include torsional angles and bond angles of prolines. Each MCM trajectory was terminated when 2,000 consecutive energy minimizations did not decrease the energy of the apparent global minimum. Besides the full-fledged model of hNa_v_1.5, we used reduced models involving only VSD-III and repeat IV of PD (PD-IV), which is proximal to VSD-III.

Cryo-EM structures of sodium channels were 3D aligned by minimizing the root mean square deviations of C^α^ atoms in the P1 helices from the crystal structure of potassium channel K_v_1.2-K_v_2.1 (PDB ID: 2R9R), the first eukaryotic P-loop channel whose crystal structure was obtained at a rather high resolution of 2.4 Å (Long et al., 2007).

To maintain the experimental template folding in the channel models, we used “pin” constraints. A pin is a flat-bottom parabolic function that contributes to the model total energy. The pin allows an alpha carbon of an amino acid residue to deviate up to 1 Å from the respective template position without a penalty and penalizes larger deviations. For all constraints, the energy penalty was calculated with force constant of 10 kcal mol^−1^ Å^−2^.

Electrostatic potential at solvent-accessible surfaces of VSD-III and PD-IV were visualized using Poisson–Boltzmann calculations with the adaptive Poisson–Boltzmann solver (APBS) plugin for PyMol (Baker et al., 2001). Atomic charges and radii were generated by the PDB2PQR server (Dolinsky et al., 2004). The models were visualized using the PyMol Molecular Graphics System, version 0.99rc (Schrödinger, New York, NY).

### *In Silico* Deactivation of VSD-III

Cryo-EM structures of the rNa_v_1.5 channel in the apo- and toxin-bond forms (Jiang et al., 2021) show that the toxin-induced deactivation of VSD-IV causes large shifts of helix IVS4 and significant conformational changes in loop IVS3-S4 and C-terminal part of helix IIIS3, but rather small structural changes in other segments of VSD-IV (Figure 2D). Based on these data, we pinned C^α^ atoms in helices IIIS1 and IIIS2 and the cytoplasmic half of helix IIIS3, while other parts of the channel were free to move. The cryo-EM structure of hNa_v_1.5 (PDB ID: 6lqa), where all VSDs are activated, was used as the starting conformation for the *in silico* deactivation. C^α^ atoms of five basic residues in helix IIIS4 were forced to move through 21 sets of planes (5 planes for five C^α^ atoms in each set), which were normal on the pore axis. Two adjacent planes were distant from one another by 0.5 Å. At each step of the *in silico* deactivation, the C^α^ atoms of basic residues were allowed to move within the corresponding plane, but not to leave it. The MCM trajectory at each step of the deactivation protocol was terminated when the last 200 consecutive energy minimizations did not improve the apparent global minimum obtained at the respective step. The starting conformation for the next deactivation step corresponded to the MC-minimized conformation of the previous step. During these calculations, the salt bridge between E1295 and R1739 was biased by a distance constraint. A similar methodology was recently used to *in silico* deactivate VSD-II in an insect sodium channel with a scorpion toxin (Zhorov et al., 2021).

## Results and Discussion

### Interface Between IVP2-S6 and VSD-III in Cryo-EM Structures of Eukaryotic Sodium Channels

**FIGURE 1:**
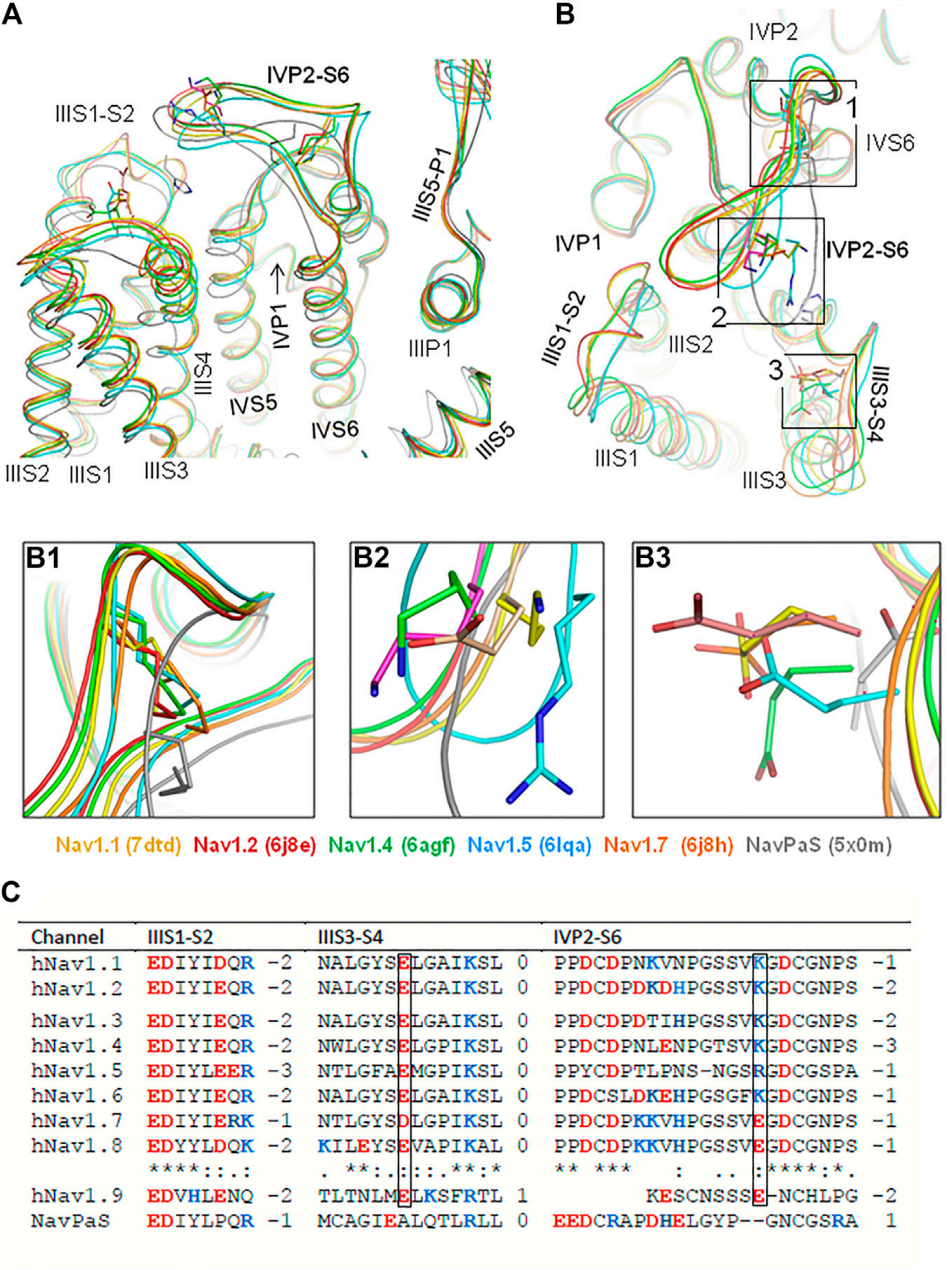
In cryo-EM structures of six eukaryotic sodium channels, extracellular loop IVP2-S6 hangs over VSD-III. **(A, B)**. Intra-membrane **(A)** and extracellular **(B)** views of the cryo-EM structures superimposed by minimizing the root mean square deviations of C^α^ atoms in the P1 helices from the crystal structure of the channel Kv1.2-Kv2.1. Note a conserved geometry of the P1 and P2 helices and rather conserved geometry of the transmembrane helices. In all the structures, loop IVP2-S6 hangs over VSD-III implying the functional significance of interdomain interactions. **(B1)**. Disulfide bonds that fasten loop IVP2-S6. **(B2).** Ionizable residues in loop IVP2-S6. **(B3)**. Acidic residues in loop IIIS3-S4. **(C).** Sequence alignment of loops IIIS1-S2, IIIS3-S4, and IVP2-S6. Residues, which are homologous to E1295 and R1739 in the hNav1.5 channel, are boxed.

Currently, 19 cryo-EM structures of eukaryotic Na_v_s are deposited in the Protein Data Bank. Figures 1A,B show the interface between VSD-III and PD-IV in the 3D aligned structures of six channels: hNa_v_1.1 (Pan et al., 2021), hNa_v_1.2 (Pan et al., 2019), hNa_v_1.4 (Pan et al., 2018), hNa_v_1.5 (Li et al., 2021), and cockroach channel Na_v_PaS (Shen et al., 2017). In these (and all other cryo-EM structures of eukaryotic Na_v_s), loop IVP2-S6 hangs over VSD-III. Geometry of the transmembrane helices and P1 and P2 helices is rather conserved, whereas extracellular loops IIIS1-S2, IIIS3-S4, and IVP1-P2 are more structurally diverse. In four channels, loop IVP2-S6 closely approaches loop IIIS1-S2, but in Na_v_1.5 and especially in Na_v_PaS, the loop is closer to IIIS3-S4, although direct contacts between the loops are lacking (Figure 1B).

Sequences of loops IIIS1-S2, IIIS3-S4, and IVP2-S6 are conserved in channels hNa_v_1.1–hNa_v_1.8 (Figure 1C). An exception is channel hNa_v_1.9, which has a short linker IVP2-S6 and unusually slow inactivation (Dib-Hajj et al., 2002; Zhou et al., 2017). In mammalian Na_v_s, the loop stems are fastened by a disulfide bond (Figure 1B1). The loop stems contain proline residues (Figure 1C), which may work as hinges during conformational transitions in this region. A conserved C-terminus of loop IVP2-S6 (GDCGNPS) is preceded by Lys or Arg in most channels. However, channels hNa_v_1.7 and hNa_v_1.8 have Glu in respective positions (Figure 1B2). Nevertheless, the net charge of the loop is negative in all the Na_v_1.x channels (Figure 1C). The basic residue in loop IVP2-S6 of channels Na_v_1.1–Na_v_1.6 may experience electrostatic attraction to highly conserved glutamate in the middle of loop IIIS3-S4 (Figure 1B3). In the cryo-EM structures of channels hNa_v_1.5 (PDB ID: 6lqa) and Na_v_PaS (PDB ID: 5×0m), the distance between the basic and acidic residues is much shorter than in other channels.

**FIGURE 2:**
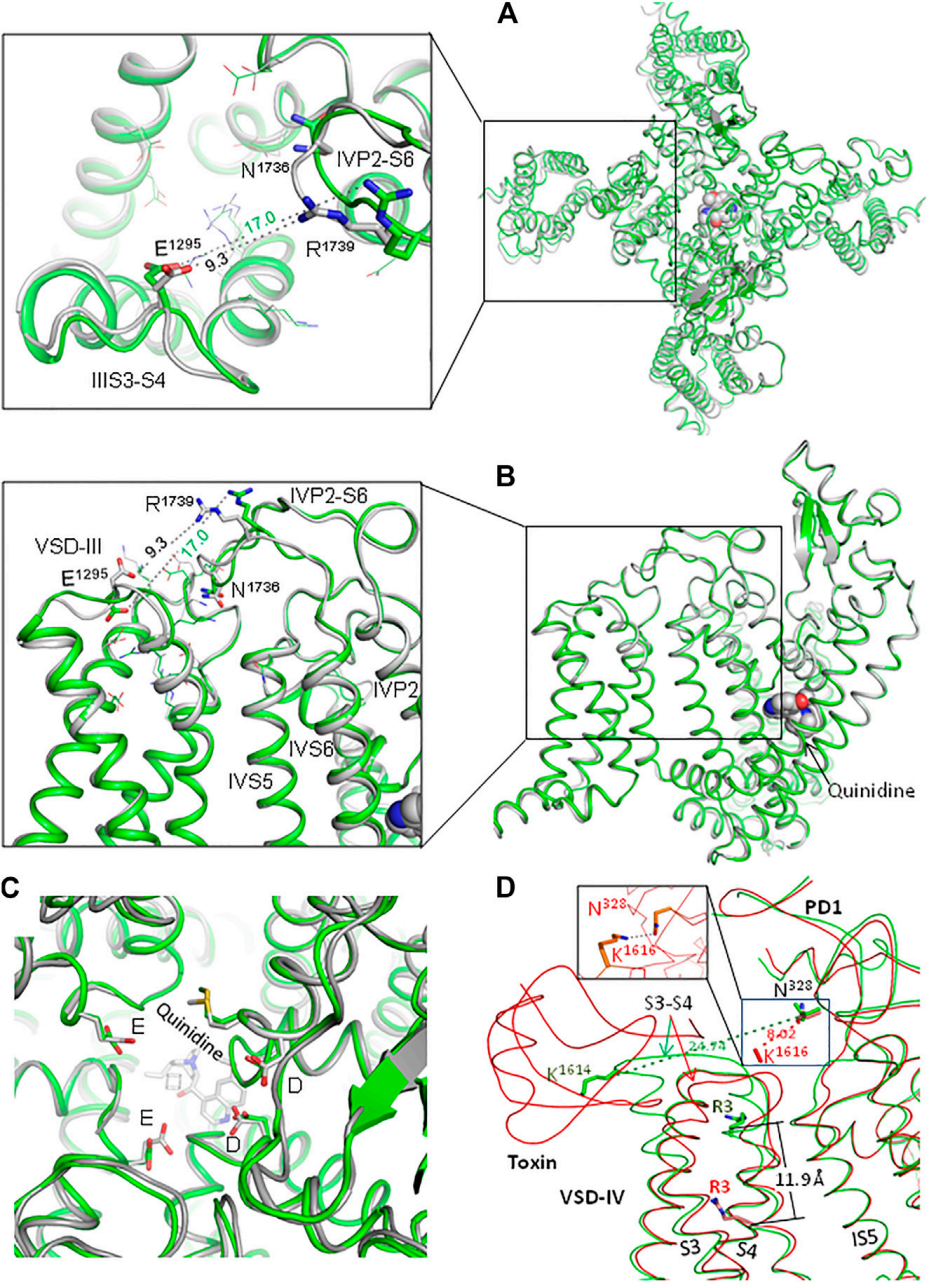
Extracellular loops in two cryo-EM structures of channel hNa_v_1.5. **(A, B)**. Extracellular **(A)** and side **(B)** views of hNa_v_1.5 in the apo-form (green; PDB ID: 7dtc) and in complex with quinidine (gray, PDB ID: 6lqa). Note different conformations of loops IIIS3-S4 and IVP2-D6 and very different distances between E1295 and R1739. **(C)**. Extracellular view of the outer pore. Note different conformations of the outer carboxylates (EEDD ring). **(D)** Cryo-EM structures of the rNa_v_1.5 channel in the apo form (green; PDB ID: 6uz3) and in complex with the deathstalker scorpion toxin (red; PDB ID: 7k18). The toxin shifts down IVS4; the distance between C^β^ atoms of arginine R3 in the two states is 11.9 Å. Loop IVS3-S4 moves much closer to loop IS5-P1 so that the distance between C^β^ atoms of K1614 and N328 decreases from 24.7 to 8.0 Å (hNa_v_1.5 numbering). At the same time, helices IVS1, IVS2, and IVS3 undergo only small shifts upon binding of the toxin: on average, C^α^ atoms of these helices are shifted by ∼ 1 Å (not shown). In the MC-minimized model with rigid backbones, K^1614^ and N^328^ are H-bonded (inlet).

In two cryo-EM structures of channel hNa_v_1.5, conformations of loop IVP2-S6 are rather different (Figures 2A,B). Conformations of the outer carboxylates (ring EEDD) are also rather different (Figure 2C). In quinidine-bound channel Na_v_1.5 (PDB ID: 6lqa), the distance between E1295 and R1739 (9.3 Å) is almost twice as small as in the apo-hNa_v_1.5 (PDB ID: 7dtc). In the interface between VSD-III and loop IVP2-S6, the quality of cryo-EM structure 6lqa is higher than that of 7dtc (Supplementary Figure S1). The cryo-EM structure of channel rNa_v_1.5 shows PD in presumably inactivated state and VSDs in partially activated states (Jiang et al., 2020). 3D-aligned structures of hNa_v_1.5 and rNa_v_1.5 are very similar (not shown), implying that both channels are frozen in the same state. However, it is unclear whether the apo- and flecainide-bound channels are captured in functionally different inactivation states.

Experimental structures of eukaryotic channels with deactivated VSD-III are lacking. However, cryo-EM structures of rNa_v_1.5 in the apo-form and in complex with a deathstalker scorpion toxin (Jiang et al., 2021) show that toxin-induced deactivation of VSD-IV dramatically downshifted IVS4 and caused large conformational changes in loop IVS3-S4 (Figure 2D). Importantly, loop IVS3-S4 moved much closer to loop IS5-P1. The above data suggest that the voltage-dependent deactivation of VSD-III also may dramatically decrease the distance between loops IIIS3-S4 and IVP2-S6.

### Interface Between VSD-III and IVP2-S6 in hNa_v_1.5 Is a Hot Spot of Mutations Associated With Cardiac Arrhythmias

**FIGURE 3:**
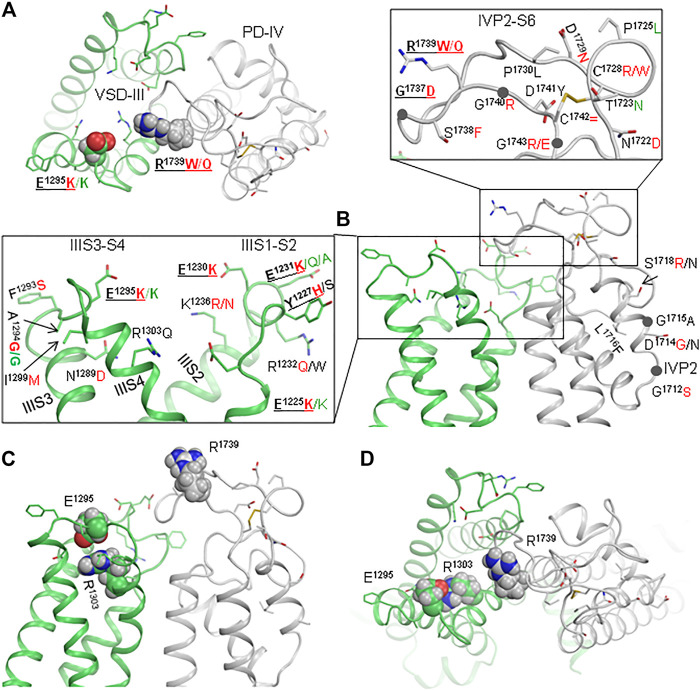
Interface between VSD-III and IVP2-S6 is a hotspot of mutations associated with Brugada syndrome. **(A, B)** Extracellular and side views of the cryo-EM structure of quinidine-bound channel hNav1.5 (6lqa). Enlargements of panel **(B)** show residues whose disease variants are reported in ClinVar. Variants associated with BrS and LQTS are indicated by red and green letters, respectively. **(C, D)** Side and extracellular views of the cryo-EM structure of hNav1.5 in the apo-state (7dtc).

Cryo-EM structures show that the interface is flexible (Figures 2A,B, and 3C,D). Genetic variants of many residues in loops IVP2-S6, IIIS1-S2, and IIIS3-S4 and helix IVP2 are associated with arrhythmias (Figures 3A,B). Lysine substitutions E1225K, E1230K, E1231K, and E1295K, which reverse the residue charge, as well as most of the other mutations shown in enlargements of Figure 3B, would affect electrostatic interactions between VSD-III and loop IVP2-S6. The above data motivated us to generate mutants E1295R, R1739E, N1736R, and E1295R/R1739E and explore their electrophysiological characteristics.

### Biophysical Characteristics of the WT and Mutant Channels

**FIGURE 4:**
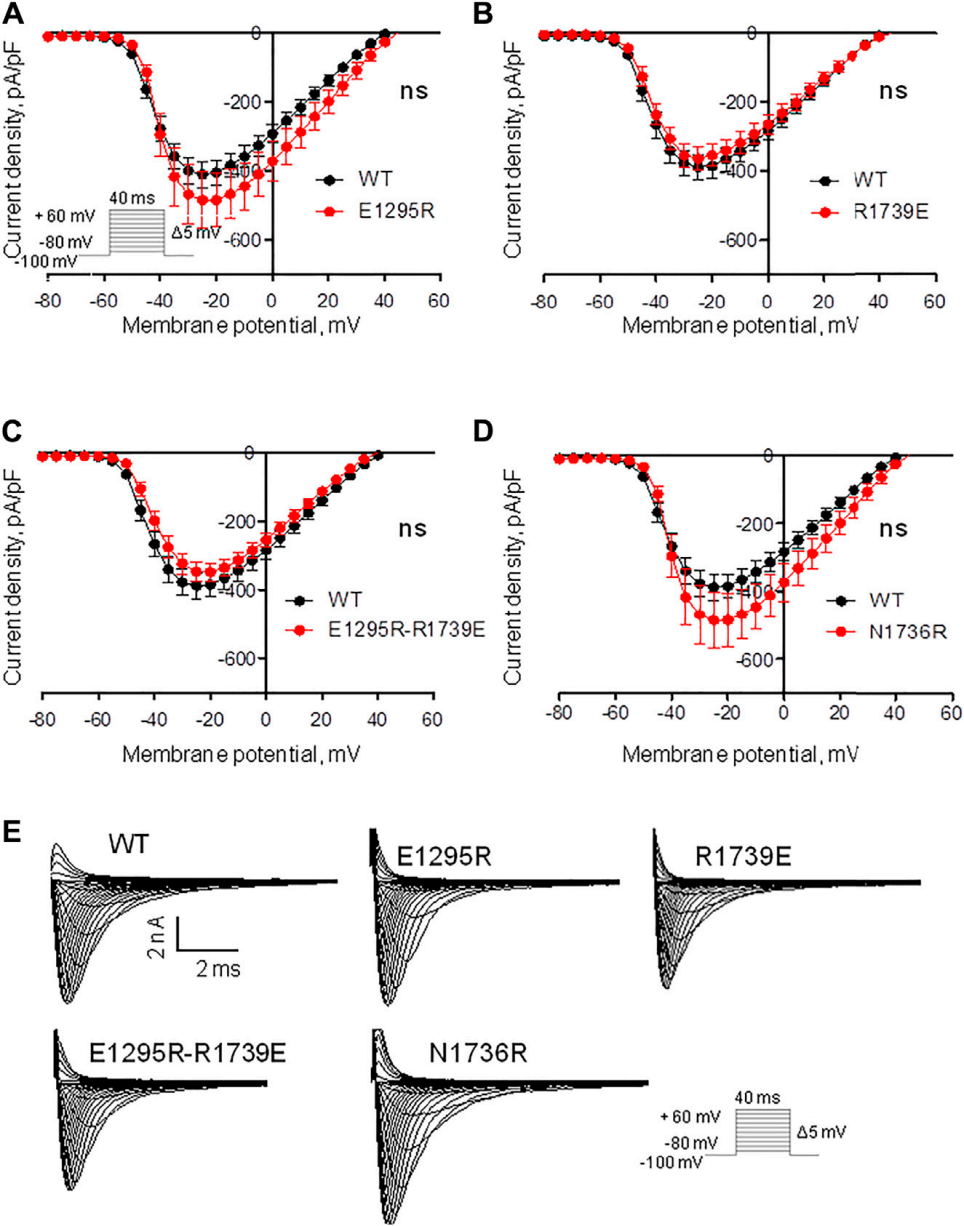
Mutations have a small impact on the voltage dependence of sodium current density. (See Supplementary Table S1 for numerical data.) **(A)** Current density of mutant E1295R insignificantly increased vs. the WT channel. **(B, C)** Current density of mutant R1739E and double mutants E1295R/R1739E is similar to that of WT channel. **(D)** Current density of mutant N1736R insignificantly increased vs. the WT channel. **(E)** Examples of current traces for the WT and mutant channels.

We expressed the WT and mutant channels in the HEK-293T expression system. No significant changes were observed in the peak sodium current of mutants vs. the WT channel (Figures 4A–D and Table 1), suggesting that the mutations did not affect protein trafficking or assembly.

**FIGURE 5:**
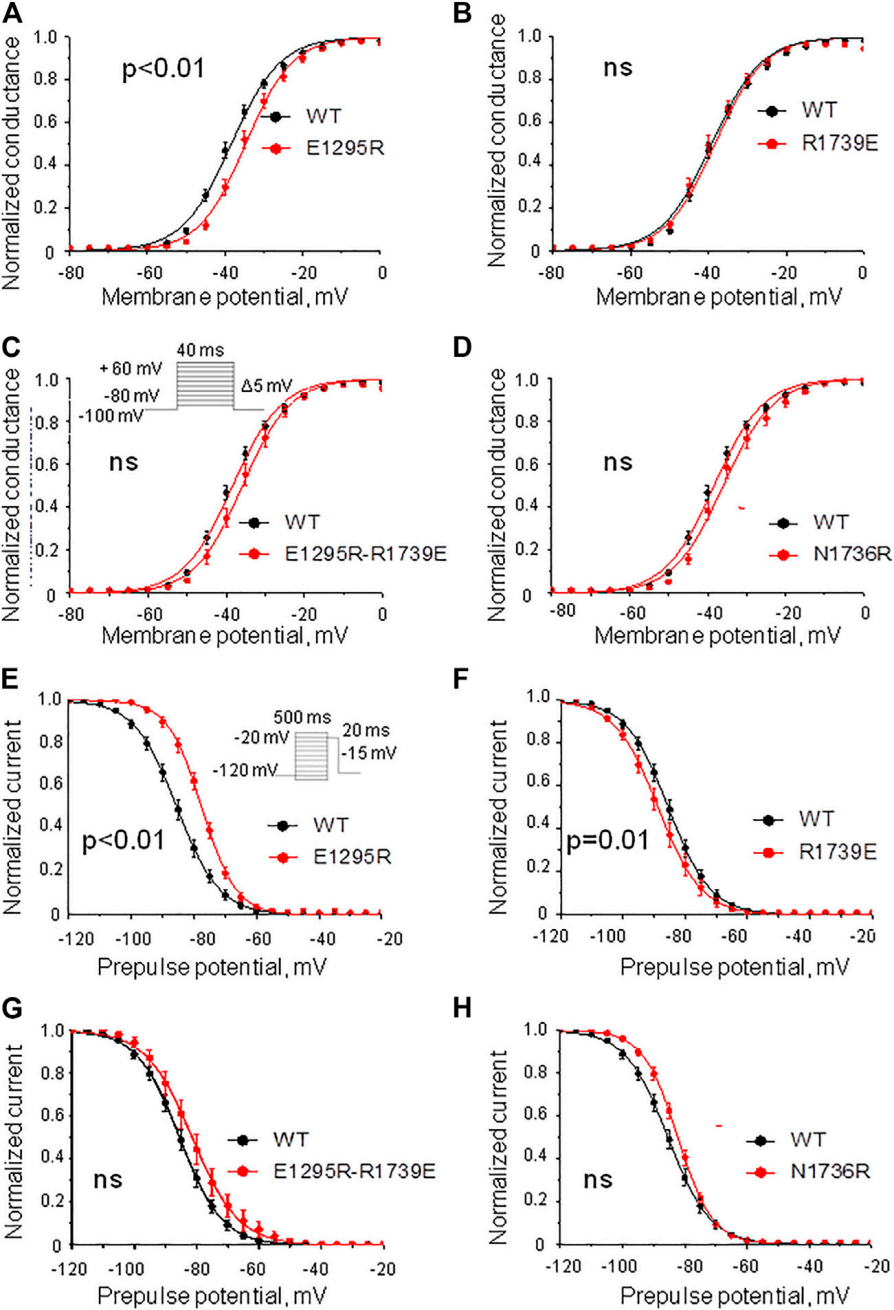
Voltage dependence of activation and steady-state inactivation. The least-square fits to the Boltzmann function for the WT channel (black) and mutant channels (red) are shown. **(A–D)** Voltage dependence of steady-state activation. **(A)** Substitution E1295R impaired the activation. **(B)** Mutation R1739E did not affect the activation. **(C,D)** Double mutation E1295R/R1739E **(C)** and mutation N1736R **(D)** insignificantly changed the activation. **(E–H)** Steady-state inactivation. **(E)** Mutation E1295R impaired the inactivation. **(F)** Mutation R1739E slightly facilitated the inactivation. **(G,H)** Double mutation E1295R/R1739E **(G)** and mutation N1736R **(H)** insignificantly impaired the inactivation.

Mutant channel E1295R demonstrated a depolarizing shift of 4 mV in the half voltage of activation (Figure 5A, Table 1). No statistically significant changes of steady-state activation were observed for mutant channels R1739E (Figure 5B, Table 1), N1736R (Figure 5D, Table 1), or E1295R/R1739E (Figure 5C, Table 1), indicating that the depolarizing shift of steady-state activation in mutant channel E1295R was rescued by double mutation E1295R/R1739E. All mutant channels demonstrated typical normalized current traces (Figure 4E). The half voltage of steady-state inactivation in mutant channel E1295R was shifted in the depolarizing direction by 7.6 mV (Figure 5E, Table 1). Mutant channel R1739E demonstrated a small but statistically significant hyperpolarizing shift of the steady-state inactivation (Figure 5F, Table 1). No statistically significant change of the steady-state inactivation was observed for the double mutant channel E1295R/R1739E (Figure 5G, Table 1) or mutant channel N1736R (Figure 5H, Table 1).

**FIGURE 6:**
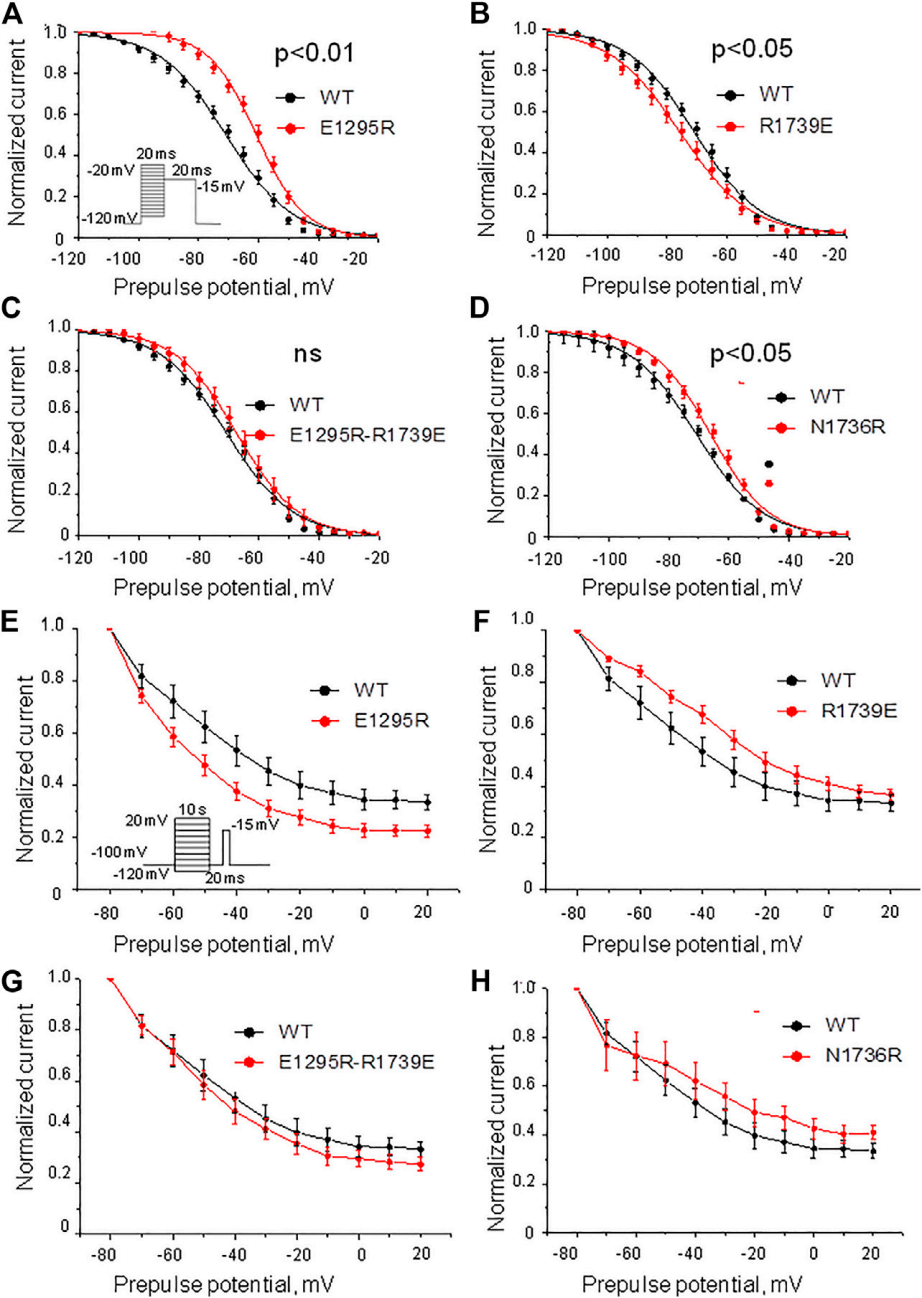
Voltage dependence of the steady-state fast and slow inactivation. **(A–D)** Voltage dependence of the steady-state fast inactivation. Shown are least-square fits to the Boltzmann function. **(A)** Mutation E1295R impaired the inactivation. **(B)** Mutation R1739E slightly enhanced the inactivation. **(C)** The voltage dependence of inactivation in the double mutant E1295R/R1739E is similar to that in the WT channel. **(D)** Mutations N1736R caused a positive shift of the inactivation curve. **(E–H)** Voltage dependence of the steady-state slow inactivation. Comparison was performed using I_Na_ at 20 mV. Characteristics of the WT channel: I_Na_/I_max_ = 0.32 ± 0.02, *N* = 11. **(E)** Mutation E1295R slightly enhanced the steady-state slow inactivation (I_Na_/I_max_ = 0.23 ± 0.02; *p* = 0.0074, N = 12). **(F)** Mutation R1739E did not significantly affect the steady-state slow inactivation (I_Na_/I_max_ = 0.37 ± 0.02; *p* = 0.15, *N* = 8). **(G)** Double mutation E1295R/R1739E slightly enhanced the steady-state slow inactivation (I_Na_/I_max_ = 0.28 ± 0.03; *p* = 0.36, *N* = 11). **(H)** Mutation N1736R slightly retarded steady-state slow inactivation (I_Na_/I_max_ = 0.18 ± 0.05; *p* = 0.037, *N* = 7).

Mutation E1295R caused a depolarizing shift of ∼ 10 mV in the half voltage of steady-state fast inactivation (Figure 6A, Table 1). Mutation R1739E enhanced the steady-state fast inactivation with ∼5 mV hyperpolarizing shift of the half voltage (Figure 6B, Table 1). The steady-state fast inactivation of the double mutant E1295R/R1739E was similar to that in the WT channel (Figure 6C, Table 1). Mutant channel N1736R hindered the steady-state fast inactivation, demonstrating a ∼ 4 mV positive shift (Figure 6D and Table 1).

Mutation E1295R significantly enhanced the steady-state slow inactivation (Figure 6E), whereas mutation R1739E impaired it (Figure 6F). In double mutant E1295R/R1739E, the voltage dependence of steady-state slow inactivation was similar to that of the WT channel. Mutation N1736R apparently impaired slow inactivation (Figure 6H), but the effect was statistically insignificant.

The above data indicate the functional importance of residues E1295 and R1739. However, since point mutations E1295R and R1739E shifted the voltage dependence curves in different directions, the rescue effect of the double mutant may indicate additive effects of each substitution. The structural interpretation of the experimental data is complicated because E1295 and R1739 are rather close to other ionizable residues in VSD-III and loop IVP2-S6 (Figure 1C, Figure 2B). To further explore possible interactions between VSD-III and loop IVP2-6, we employed computational methods as described below.

### *In Silico* Deactivating VSD-III With Salt Bridge E1295-R1739

Comparison of the cryo-EM structures of channel rNa_v_1.5 and its complex with a deathstalker scorpion toxin (Jiang et al., 2021) shows a large toxin-induced downshift of helix IVS4 and significant shift of loop IVS3-S4 towards PD-IV (Figure 2D). In this channel, extracellular loop IP2-S6 with N329 at the loop apex hangs over loop IVS3-S4 with K1616 at the loop apex. The distance between C^β^_K1616 and C^β^_N329 decreases from 27.7 Å in the apo structure (PDB ID: 6uz3) to 8.0 Å in the structure with toxin-induced deactivated state of VSD-IV (PDB ID: 7k18). The side chain of K1616 is unresolved in the toxin-bound channel, but molecular modeling predicts its state-dependent H-bond with N329 (inlet in Figure 2D). At the same time, deactivation of VSD-IV resulted in minimal shifts of helices IVS1, IVS2, and IVS3. This spatial disposition of loops IP2-S6 and IVS3-S4 resembles that of loops IVP2-S6 and IIIS3-S4. Binding of protoxin-II from the Peruvian green velvet tarantula to chimeric channel Na_v_PaS/Na_v_1.7-VSD-II (Xu et al., 2019) and binding of modified huwentoxin-IV from the Chinese bird tarantula to chimeric channel Na_v_Ab/Na_v_1.7-VSD (Wisedchaisri et al., 2021) also caused large toxin-induced downshifts of helix IIS4 and rearrangements in loop IIS3-S4.

**FIGURE 7:**
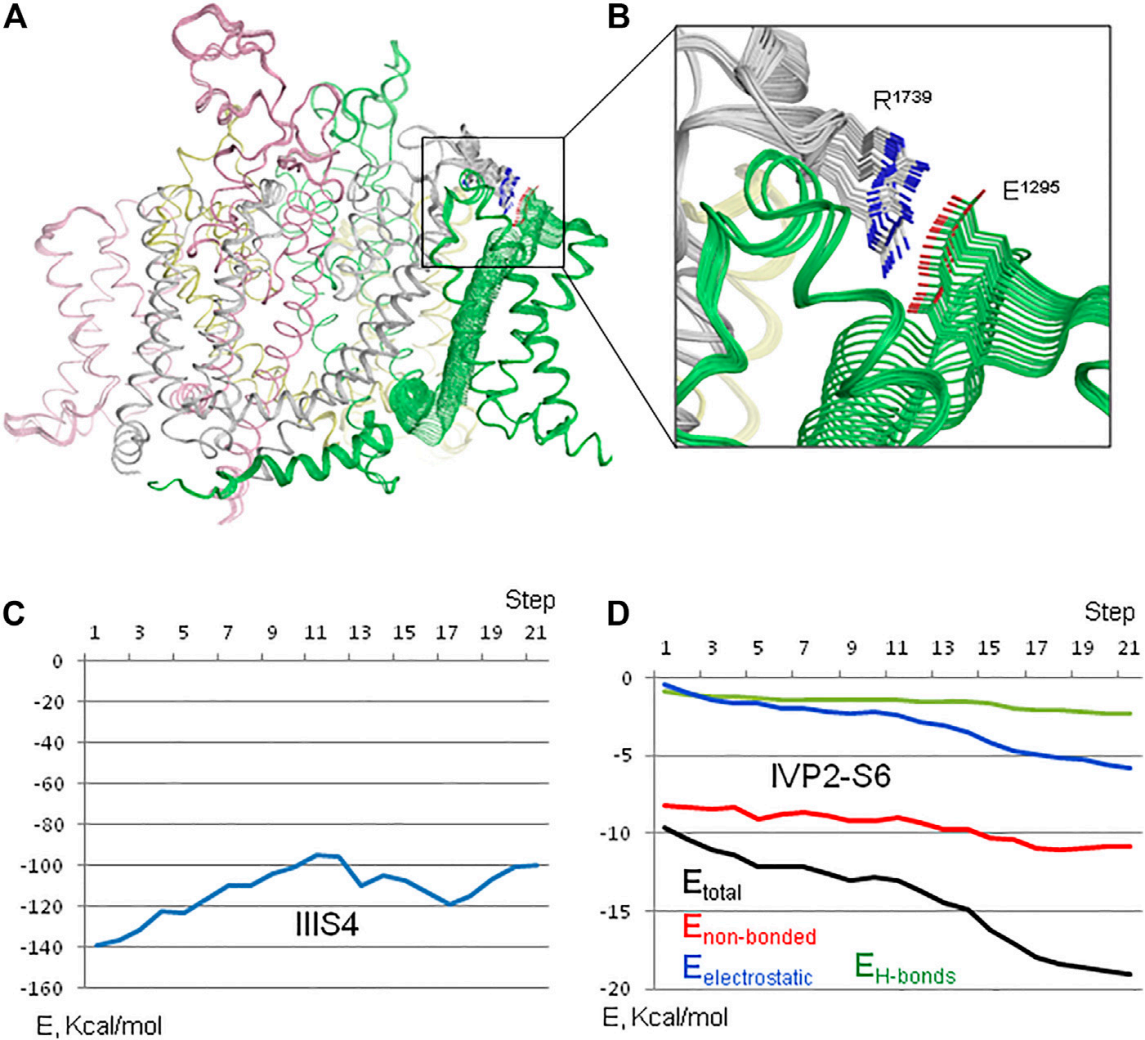
*In silico* deactivation of VSD-III. **(A)** Intra-membrane view of the channel. **(B)** Enlarged view of the constraint-biased salt bridge R1739-E1295. E1295 in linker IIIS3-S4 moves significantly with IIIS4 and R1739 follows it. **(C)** Interaction energy of IIIS4 with the rest of the channel. **(D)** Interaction energy (E_total_) and its components between loop IVP2-S6 (residues 1722–1743) and VSD-III. The energy of flanking residues in the loop stems is not counted.

**FIGURE 8:**
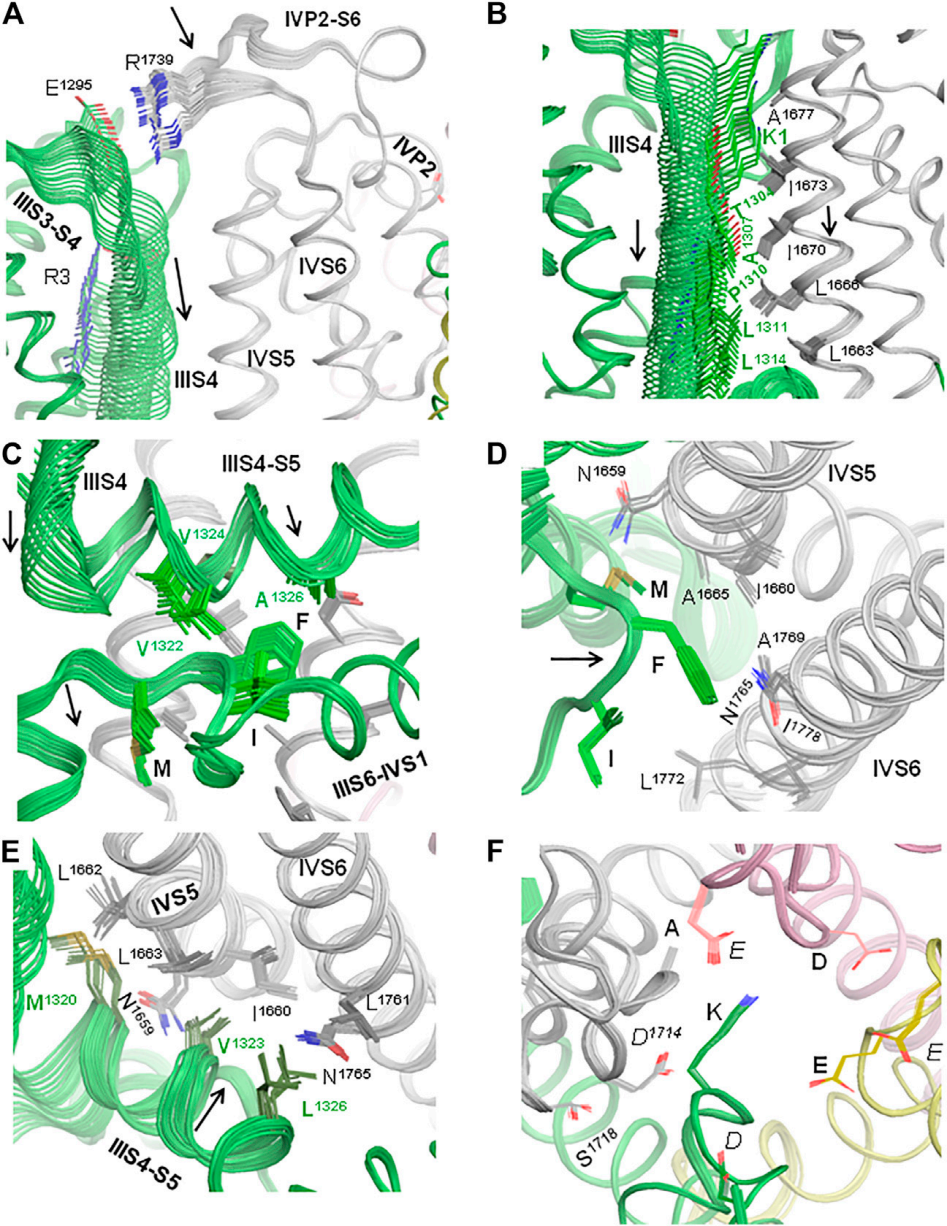
*In silico* deactivation of VSD-III. **(A)** The downshift of helix IIIS4 significantly moves loop IIIS3-S4 and loop IVP2-S6 due to the constraint-biased salt bridge R1295-R1739. Helices IVP2 and IVS6 at the N- and C-termini of the loop are also disturbed, although less significantly than the loop *per se*. **(B)** The downshift of helix IIIS4 moves helix IVS5. As IIIS4 slides over IVS5, five contacts between IIIS4 and IVS5 are switched, but due to the hydrophobic nature of residues in the interface, the interdomain friction would be small. **(C)** The downshift of the N-terminal part of linker-helix IIIS4-S5 moves the inter-repeat linker III-IV and affects contacts of hydrophobic residues in helix IIIS4-S5 with the fast inactivation tripeptide IFM. **(D)** Motions of IVS5, IVS6, and IIIS3-S4 affect their contacts with the IFM fast inactivation tripeptide. **(E)** Motions of IVS5, IVS6, and IIIS3-S4 affect contacts between these helices. **(F)**. Side chains of the outer carboxylates (*EEDD*) shift more than those of the selectivity filter DEKA ring.

Membrane hyperpolarization should cause a large downshift of IIIS4 coupled with the movement of loop IIIS3-S4. Residues E1295 in IIIS3-S4 and R1739 in IVP2-S6 are rather far from each other in the cryo-EM structures (Figure 2A). However, MC minimization of the quinidine-bound hNa_v_1.5 (PDB ID: 6lqa) with the constrained salt bridge E1295-R1739 yielded a model where no C^α^ atom deviated more than 1 Å from the cryo-EM structure. To explore if salt bridge E1295-R1739 may retain in the hyperpolarized membrane, we *in silico* deactivated VSD-III (see Methods) with the constrained salt bridge. The downshift of IIIS4 caused large changes in loops IIIS3-S4 and IVP2-S6 (Figures 7A,B and 8A), in linker-helix IIIS4-S5, and in inter-repeat linker III/IV that includes the fast inactivation tripeptide IFM (Figure 8C) and some downshift of IVS5 (Figure 8B). Sliding IIIS4 over IVS5 switched multiple interdomain contacts, but a greasy interface between these helices would minimize the friction. Significant alterations of mainly hydrophobic contacts are seen in interfaces IIIS4-S5/IVS5 and IIIS4-S5/IVS6 (Figure 8E) and between the IFM fast inactivation tripeptide and helices IIIS4-S5, IVS5, and IVS6 (Figures 8C,D). Helix IVS6 shifted with IVS5 and IIIS4-S5 (Figure 8E), implying a pathway for the motion transmission between VSD-III and the IFM binding residues in IVS6. Another pathway for transmission of the IIIS4 motion may involve loop IVP2-S6, which is linked to helix IVS6 (Figure 8A). The VSD-III induced deviation of loop IVP2-S6 caused a noticeable disturbance at the selectivity filter region (Figure 8F), suggesting a pathway for the motion transmission from VSD-III to the SF gate.

The interaction energy of helix IIIS4 with the rest of the channel increased upon VSD-III deactivation (Figure 7C) in agreement with the notion that in the absence of hyperpolarizing membrane potential, the activated states of VSDs are more preferable than deactivated states. However, the interaction energy of loop IVP2-S6 with the rest of the channel decreased (become more favorable) with the downshift of IIIS4 (Figure 7D), supporting a possibility of salt bridge E1295-R1739 in the resting VSD-III. We suggest that during cycles of membrane depolarization/repolarization, loop IVP2-S6 would shuttle with IIIS4, disturbing the selectivity filter region, which would finally switch to the state with a closed SF gate.

### State-Dependent Electrostatic Potentials at Interface VSD-III/PD4

**FIGURE 9:**
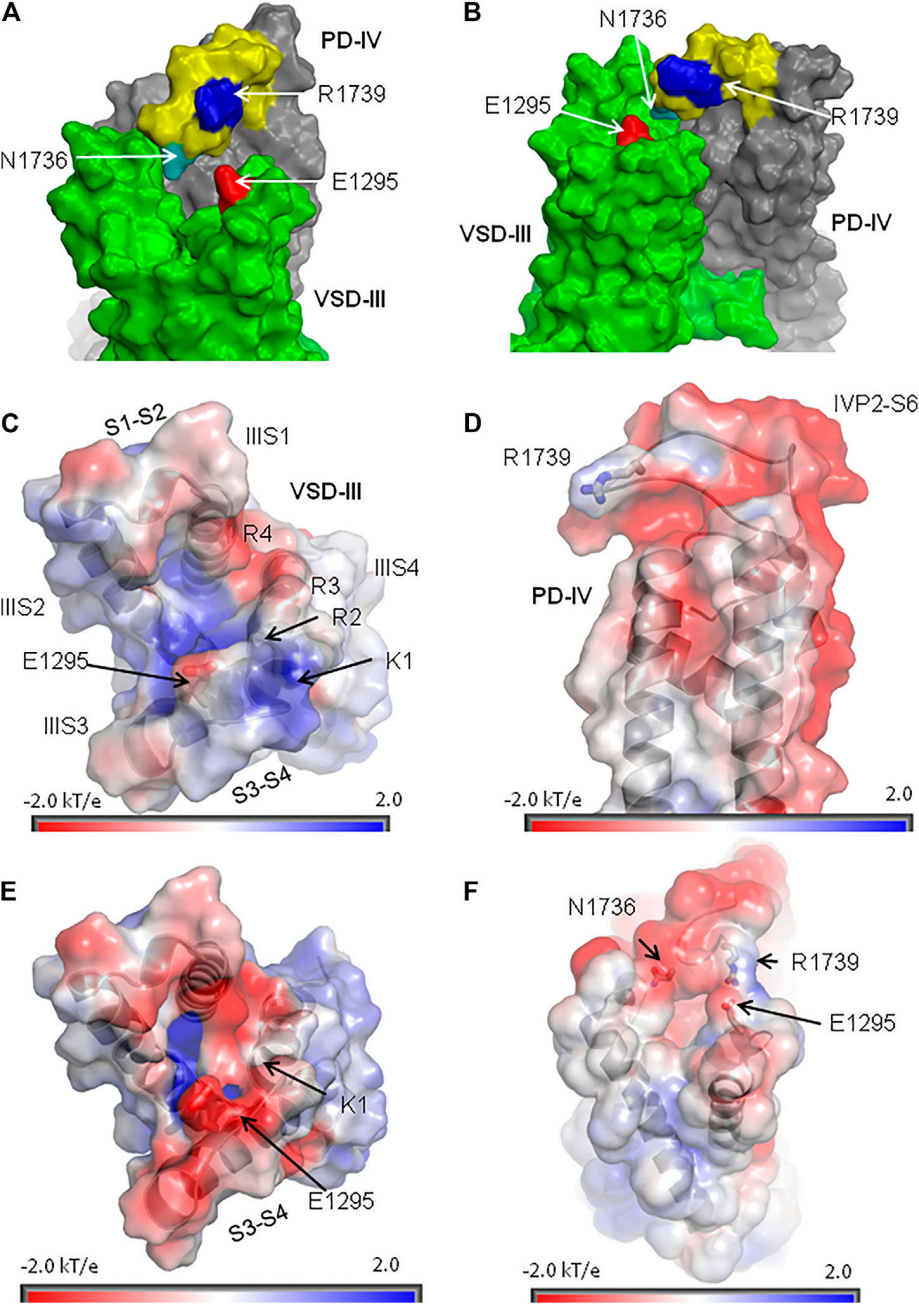
Extracellular loop IVP2-S6 hangs over VSD-III. **(A, B)** Orthogonal views of VSD-III and PD-IV. Loop IVP2-S6 (yellow with blue R1739 and cyan N1736) approaches linkers IIIS1-S2 and IIIS3-S4 but does not establish contacts with E1295 (red). **(C)** Extracellular view at the map of electrostatic potential plotted on the solvent-accessible surface of VSD-III in the cryo-EM structure (PDB ID: 6lqa). The positive potential (blue) at the extracellular surface of VSD-III is due to basic residues in IIIS4. **(D)** Side view at the map of electrostatic potential plotted at the solvent-accessible surface of PD-IV. Note a positively charged R1739 on otherwise negatively charged loop IVP2-S6. **(E)** Extracellular view at the map of electrostatic potential plotted on the solvent-accessible surface of the *in silico* deactivated VSD-III. The shift of IIIS4 in the intracellular direction reduced the positive potential at the extracellular side of VSD-III, making it more attractive for R1739. **(F)** Side view of the electrostatic potential map plotted at the solvent-accessible surface of the activated VSD-III bound to PD-IV. Due to the proximity of VSD-III and PD-IV, their positive and negative potentials are reduced.

R1739 is the only basic residue in loop IVP2-S6, which approaches E1295 in the activated VSD-III (Figures 9A,B). However, other ionizable residues can contribute to electrostatic interactions between IVP2-S6 and VSD-III. We used the Poisson–Boltzmann method to compute maps of the electrostatic potential at the solvent-accessible surfaces of isolated VSD-III and PD-IV and in the complex of activated VSD-III with PD-IV (see Methods). In the lone activated VSD-III, the electrostatic map at the extracellular surface of IIIS3-S4 has predominantly positive potential due to K1 and R2 with a negative-potential patch from E1295 (Figure 9C). Most of the lone PD-IV has a negative potential with the electropositive patch from R1739 (Figure 9D). The latter would be attracted by E1295 but repelled by K1 and R2. This can explain why salt bridge R1739-E1295, while sterically possible (Figures 7C,D), is not seen in the cryo-EM structures of Na_v_1.5 with the activated VSD-III. In the deactivated VSD-III, K1, R2, and other basic residues in IIIS4 are downshifted and the electrostatic potential at the IIIS3-S4 surface becomes strongly negative (Figure 9E) and more attractive to R1739. The predominantly negative surface potential in the lone PD-IV (Figure 9D) is compensated by the predominantly positive potential in the lone activated VSD-III (Figure 9C). As a result, in the model of activated VSD-III/PD-IV with salt bridge E1295-R1739, the electrostatic potential is rather neutral (Figure 9F). Thus, Poisson–Boltzmann calculations support the proposition of state-dependent salt bridge E1295-R1739.

### Modeling Channel Mutants

**FIGURE 10:**
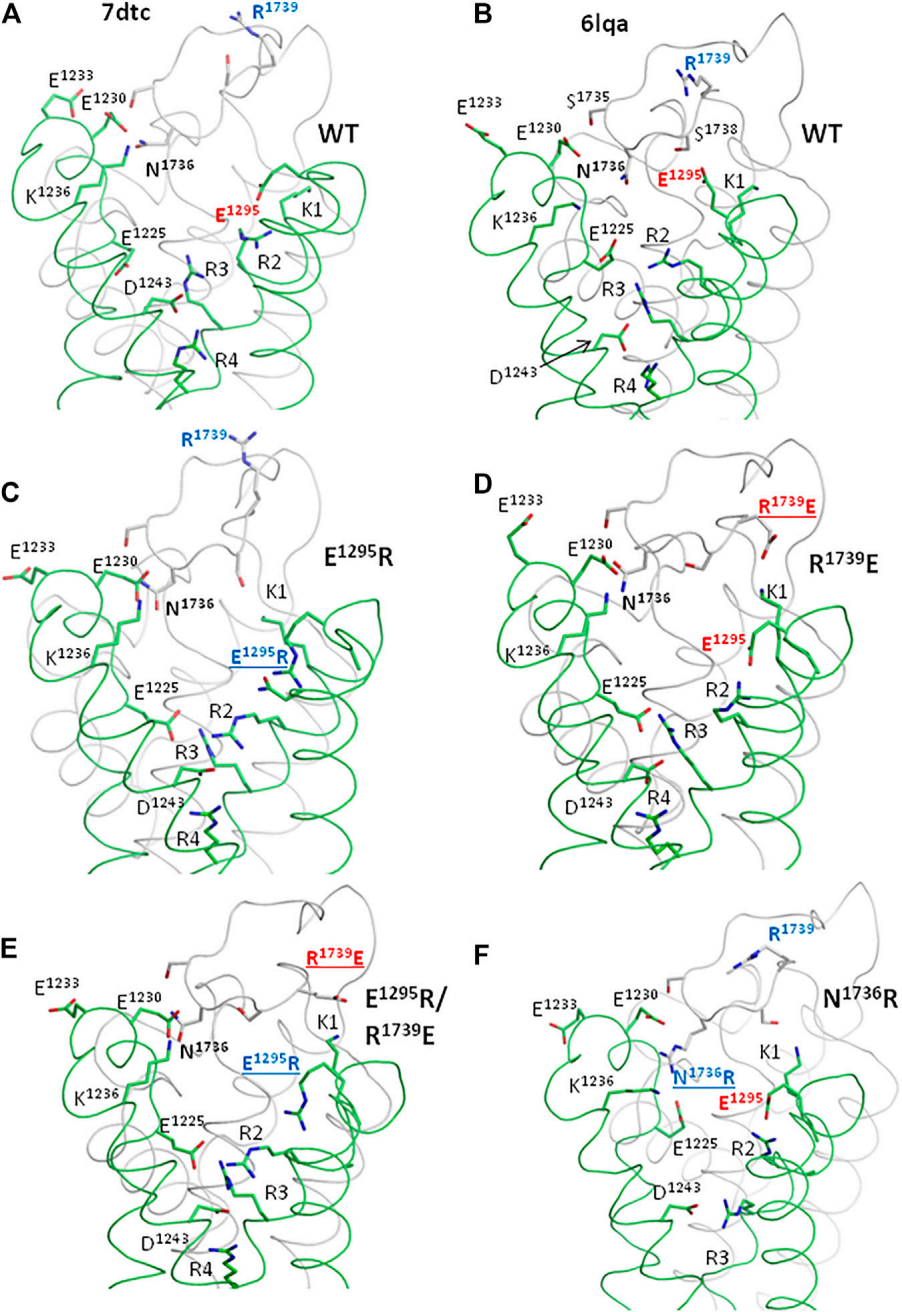
Ionizable residues and N1736 in the cryo-EM structures of hNa_v_1.5 **(A, B)** and MC-minimized models of mutants **(C–E)**. Only parts of VSD-III and PD-IV are shown for clarity. **(A, B)** Cryo-EM structures of the channel hNa_v_1.5 mutant E1784K (PDB ID: 7dtc) and hNa_v_1.5 with flecainide (PDB ID: 6lqa). Different conformations of polar residues (including E1295, R1739, and N1736) indicate their high flexibility. **(C)** Mutation E1295R decreased electrostatic attraction of R1739 to VSD-III and caused a loss-of-function phenotype. The mutation facilitated slow inactivation and retarded activation but facilitated fast inactivation **(Figures 5, 6)**. This is consistent with the data that various mutations that decrease the electrostatic attraction between VSD-III and IVP2-S6 are associated with BrS **(Figure 3)**. **(D)** Mutation R1739E, which increases the attraction of IVP2-S6 to VSD-III (note salt bridge K1-R1739), retards the slow inactivation, the effect opposite to that of mutation E1295R **(Figure 6)**. **(E)**. In the double mutant channel, R1739E is salt-bridged to K1, while E1295R turns in the intracellular direction. The changed contacts are likely responsible for the rescue effect of the double mutation on slow inactivation. **(F)** Mutations N1736R, which increases the electrostatic attraction of loop IVP2-S6 to glutamates E1225 and E1230, retards slow inactivation.

In the apo-Na_v_1.5 (Figure 10A) and quinidine-bound Na_v_1.5 (Figure 10B), residues E1295, N1736, R1739, and other polar residues at interface VSD-III/IVP2-S6 have substantially different side-chain conformations. We used the cryo-EM structure of quinidine-bound Na_v_1.5, where side chains of E1295 and R1739 are relatively close to each other, to model the four mutants explored in this study.

In mutant channel E1295R, basic groups of E1295R and R1739 are far from each other (Figure 10C). Repulsion of R1739 from E1295R, K1, and R2 would destabilize the activated state of VSD-III. This may explain the depolarizing shift in the activation G-V curve (Figure 5A). The same repulsion may also explain the facilitated steady-state inactivation (Figure 5E) and steady-state fast inactivation (Figure 6A) of the mutant. The depolarizing shift of steady-state inactivation in mutant E1295R (Figure 5E) is consistent with the same-direction shift observed for the LQT3-associated gain-of-function mutant E1295K expressed in HEK-293T cells (Abriel et al., 2001). Mutation E1295R also caused a negative shift of the voltage dependence of slow inactivation (Figure 6E). We suggest that upon a single event of VSD-III activation, repulsion of R1739 from the basic residues in IIIS4 would push IVP2-S6 in the extracellular direction, transmitting the activation signal to the SF gate (Figure 8F). Open and closed states of the SF gate should be separated by a high energy barrier. The probability of overcoming the barrier increases with the number of cycles of channel activation and deactivation. Return of the SF gate to the open state would take time.

In the model of mutant channel R1739E, salt bridge R1739E-K1 stabilized the activated state of IIIS4 (Figure 10D). The curves of the voltage-dependent activation (Figure 5B), steady-state inactivation (Figure 5F), and steady-state fast inactivation (Figure 6B) shifted in directions, which are opposite to those observed for mutant channel E1295; the effects were small but statistically significant. A likely cause is that salt bridge R1739E-K1 did not add much to the energetically preferable activated state of IIIS4 in the depolarized membrane. A relatively large hyperpolarizing shift of slow inactivation (Figure 6F) was opposite to the depolarized shift in mutant E1295R (Figure 6E) likely because salt bridge R1739E-K1 keeps loop IVP2-S6 closer to VSD-III, weakens transmission of the VSD-III activation signals to the SF gate, and thus decreases a probability of the SF gate closure.

In the model of double mutant channel E1295R/R1739E, engineered glutamate R1739E was also salt-bridged to K1, while engineered arginine E1295R was repelled by K1 and adopted conformation like in mutant E1295R (Figure 10E). The electrostatic interaction between VSD-III and IVP2-S6 was weaker than that in the mutant channel E1295R and more similar to that in the WT channel.

In model N1736R, the engendered arginine was salt-bridged with E1125 and E1230, while R2 and R3 formed salt bridges with E1295 and D1243, respectively (Figure 10F). Structural mechanisms by which these rearrangements affect channel activation and steady-state inactivation are unclear.

### Possible Mechanisms of Motions Transfer From VSD-III to the Inactivation Gates

**FIGURE 11:**
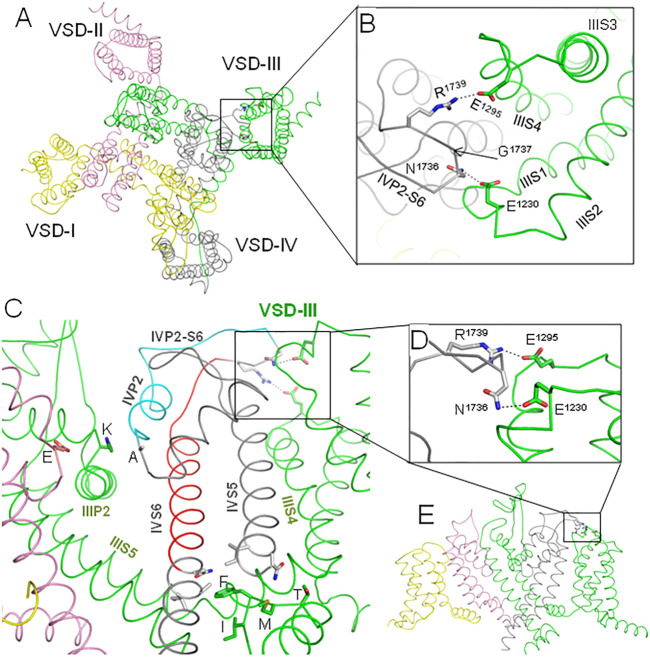
Proposed pathways of allosteric signal transmission from VSD-III to the fast and slow inactivation gates. Repeats I, II, III, and IV are yellow, pink, green, and gray, respectively. **(A)**. Extracellular view of the hNa_v_1.5 channel cryo-EM structure (PDB ID: 6lqa), which is MC-minimized with C^α^ atoms pinned to the experimental positions. **(B)** Enlarged extracellular view of the interface between VSD-III and IVP2-S6. Salt bridge R1739-E1295 was biased by a distance constraint. This decreased the distance between ^Nη^R1739 and ^Oε^E1295 from 9.3 Å in the cryo-EM structure to 3.0 Å in the MC-minimized structure. However, not a single C^α^ atom deviated by more than 1 Å from the experimental position. **(C)**. Intra-membrane view of the channel with some parts removed for clarity. Helix IVP2 and the N-terminal part of linker IVP2-S6, which are proposed to transmit allosteric signals from VSD-III to the selectivity filter gate, are cyan. Glutamate **(E)**, lysine **(K)**, and alanine **(A)** residues in the selectivity filter DEKA ring are shown by sticks. The C-terminal parts of linker IVP2-S6 and helix IVS6, which are proposed to contribute to the motion transmission from VSD-III to the fast inactivation gate, are red. Side chains of the fast inactivation tetra-peptide IFMT in the intracellular linker III/IV and some residues that may interact with the tetra-peptide are shown by sticks. **(D)** Enlarged side view of the interface between IVP2-S6 and VSD-III. **(E)** Overall intra-membrane view of the channel with some parts removed for clarity.

Results of our computations suggest that loop IVP2-S6 may transmit voltage-dependent motions of VSD-III to the alanine residue in the selectivity filter DEKA ring and to D1714 in the ring of outer carboxylates (Figure 8F). Mutations in the latter rings are known to affect slow inactivation; see (Tikhonov and Zhorov, 2007) and references therein. Mutation in the P-loop of repeat IV affects the ion selectivity and ion permeation in the Nav1.4 channel, suggesting high flexibility of this loop (Tsushima et al., 1997a; Tsushima et al., 1997b). These data are consistent with our proposition that transfer of the VSD-III motions to helix IVP2 through loop IVP2-S6 may switch the SF gate to the slow inactivation state (Figure 11).

The other end of loop IVP2-S6 may transmit motions of VSD-III to the N-terminal part of helix IVS6 (Figure 8A) and, possibly, along this helix to the IFM tripeptide (Figures 8D, 11). However, motions of IIIS4 are also transmitted to the IFM motif through helices IVS5 and IIIS4-S5 (Figures 8 B,C, 11). The degree of IVS6 contribution to the motion transmission from VSD-III to the fast inactivation gate is unclear, but our data demonstrate that E1295R retards the steady-state inactivation (Figure 5E) and steady-state fast inactivation (Figure 6A), which support the role of this pathway.

Sequences of loop IVP2-S6, which are conserved in channels Na_v_1.1–Na_v_1.8, contain 13–14 residues between disulfide-bonded cysteines (Figure 1C). An exception is channel Na_v_1.9, whose loop IVP2-S6 has only six residues between the flanking cysteines. Such a short loop is unlikely to interact with VSD-III. The Na_v_1.9 channel has unique electrophysiological properties among Na_v_1.x channels (Dib-Hajj et al., 2002), including extremely slow inactivation (Zhou et al., 2017). In view of our proposition that loop IVP2-S6 mediates the signal transmission from VSD-III to the SF gate, the ultra-slow inactivation of Na_v_1.9 may indicate that motions of VSD-III are not transmitted to the SF gate and the inactivation is triggered by another mechanism.

Our experimental data show the functional importance of residues R1739, E1295, and N1736. However, the fact that electrophysiological characteristics of the double mutant R1739E/E1295R are rather similar to those of the WT channel does not necessarily mean that R1739 and E1295 form a salt bridge in the resting VSD-III. Further experiments such as locking VSD-III in the deactivated state with disulfide or metal ion bridges are necessary to demonstrate direct interactions of these residues. Meanwhile, the possibility of the salt bridge is supported by the following indirect evidences. 1) The charge elimination in BrS-associated variants R1739Q/W or change reversal in disease variant R1295K (Figure 3B) implies the involvement of these residues in functionally important electrostatic interactions. In the cryo-EM structure 6lqa, E1295 is the closest acidic residue to attract R1739. 2) According to our computations, IVP2-S6 “enjoys” rather than resists the coupled downshift with IIIS4 (Figure 7D). 3) In the resting VSD-III, E1295 creates a cation-attractive potential for R1739 (Figure 9E). 4) Some analogy of the state-dependent contacts between VSD-III and loop IVP2-S6 may be found in the cryo-EM structures of rNav1.5, where loops IP2-S6 and IVS3-S4 are rather far from each other in the activated state of IVS4 but form a close contact in the resting state (Figure 2D). 5) The proposed mechanism explains why multiple mutations in the putative pathway of the motion transmission from VSD-III to the SF gate are associated with the loss-of-function Brugada syndrome (Figure 3B).

In conclusion, here, we generated four mutants at the interface of VSD-III with the extracellular loop IVP2-S6, expressed the mutants in HEK-293T cells and explored their electrophysiological properties. Mutation E1295R in loop IIIS3-S4 demonstrated the strongest impact on the voltage dependence of channel activation and inactivation. Based on molecular modeling, we propose that E1295 forms a state-dependent salt bridge with R1739, the only basic residue in helix IVP2-S6 that approaches E1295 in the cryo-EM structure of quinidine-bound hNa_v_1.5. We further propose that contacts between VSD-III and loop IVP2-S6 mediate transmission of the voltage-dependent motions from VSD-III to the SF gate and contribute to motion transmission from VSD-III to the fast inactivation gate. Our study suggests a possibility of allosteric signal transmission between VSD-III and the inactivation gates in the cardiac sodium channel. Mutations of many residues along the signal transmission pathways are associated with cardiac arrhythmias.

## Data Availability

The original contributions presented in the study are included in the article/Supplementary Material; further inquiries can be directed to the corresponding authors.

## Abbreviations

BrS, Brugada syndrome; K1, R2, R3, and R4, basic residues in IIIS4; LQTS, long-QT syndrome; MC, Monte Carlo; MCM, MC minimizations; Nav, voltage-gated sodium channel; PD, pore domain; SF, selectivity filter; TM, transmembrane; VSD, voltage-sensing domain.
