# Supplementary material for: Possible Interactions of Extracellular Loop IVP2-S6 With Voltage-Sensing Domain III in Cardiac Sodium Channel
Source: Front Pharmacol. 2021 Oct 14;12:742508. doi: 10.3389/fphar.2021.742508 (PMC8551724; doi:10.3389/fphar.2021.742508)
Supplement: Supplementary file 1 [file DataSheet1.PDF]

Supplementary Data

Possible Interactions of Extracellular Loop IVP2-S6 with Voltage-Sensing Domain III in Cardiac Sodium Channel

Anastasia K. Zaytseva[1,2] , Aleksandr S. Boitsov[1], Anna A. Kostareva[1,3,*] and Boris S. Zhorov [1,2,4,*]

[1] Almazov National Medical Research Centre, St. Petersburg, Russia; [2] Sechenov Institute of Evolutionary Physiology and Biochemistry, Russian Academy of Sciences, St. Petersburg; [3] Karolinska Institute, Stockholm, Sweden; [4] McMaster University, Hamilton, Canada

*Corresponding authors: zhorov@mcmaster.ca; anna.kostareva@ki.s

Table 1. Half-voltage ($V_{1/2}$, mV) and slope factor (k) of steady-state activation and inactivation

		WT	n	E1295R	n	R1739E	n	E1295R/ R1739E	n	N1736R	n
Current density at -20 mV	pA/pF	-400.5 ±37.8	34	-397.2± 32.8	29	-307.7± 46.4	19	-364.4± 27.0	16	-500.7± 81.9	15
Steady-state activation	$V_{1/2}$<br>k	-38.3±0.8<br>5.5±0.2	34	-34.6±0.9 **<br>5.1±0.2	29	-39.2±1.1<br>5.5±0.3	19	-35.4±1.6<br>4.9±0.2	16	-36.6±0.9<br>5.4±0.4	15
Steady-state inactivation	$V_{1/2}$<br>k	-85.2±1.2<br>5.5±0.1	30	-77.6±1.0**<br>4.8±0.2**	24	-88 .5±1.5*<br>5.7±0.2	19	-80.7±2.0<br>4.7±0.2**	16	-82.0±1.0<br>5.2±0.2	15
Steady-state fast inactivation	$V_{1/2}$<br>k	-70.8±1.6<br>9.6±0.3	30	-61.0±1.4**<br>7.4±0.3 **	21	-76.0±2.1*<br>10.0±0.3	19	-67.4±2.6<br>7.9±0.3**	16	-65.1±1.1*<br>9.1±0.4	15

** $p < 0.01$
* $p < 0.05$

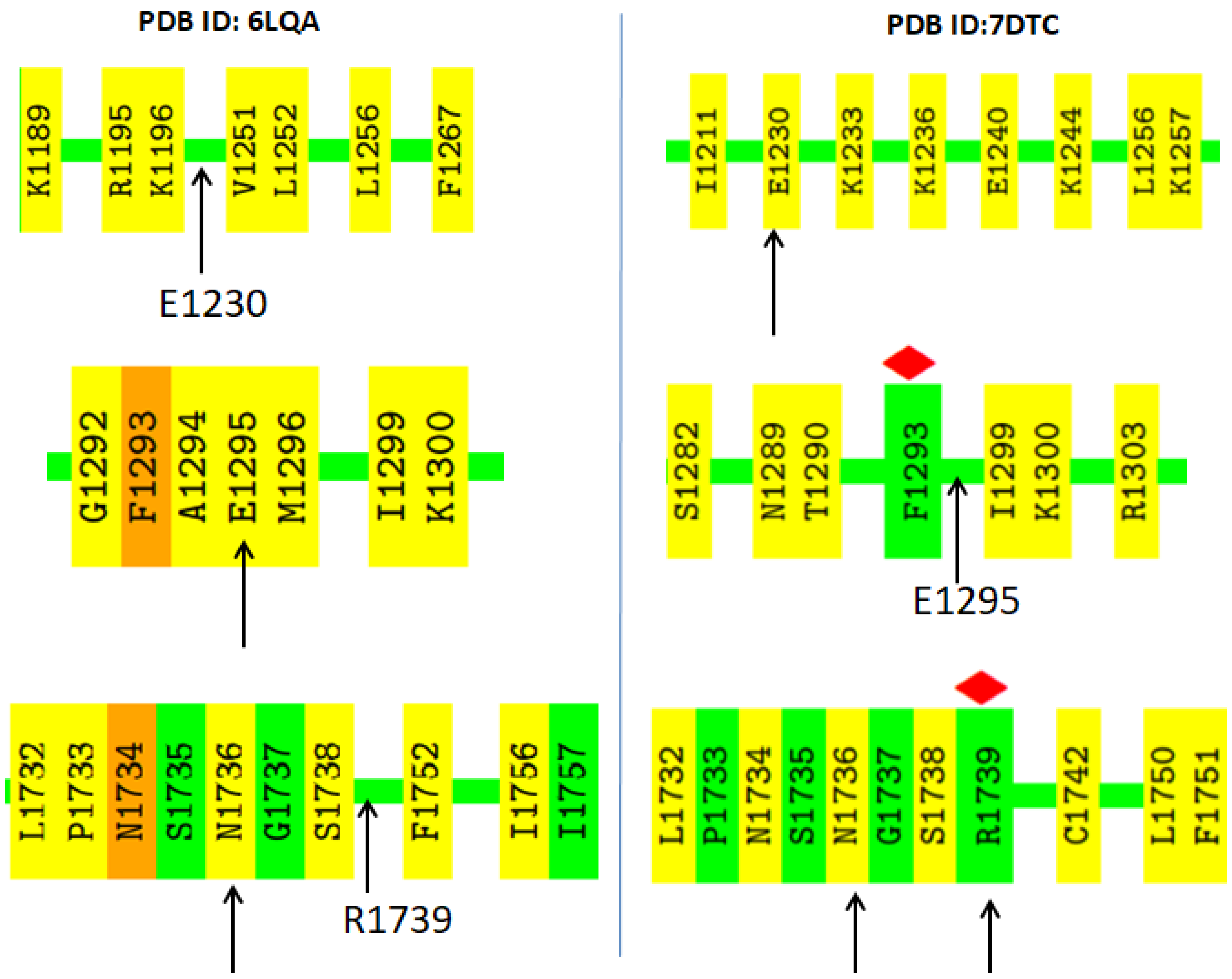

Figure S1. Quality of cryo-EM structures in the Interface between VSD-III and loop IVP2-SS6. Shown are extracts from Full wwPDB EM Validation Reports for cryo-EM structures 6lqa and 7dtc. Residues are color-coded according to the number of geometric quality criteria for which they contain at least one outlier: green = 0, yellow = 1, orange = 2 and red = 3 or more. Stretches of 2 or more consecutive residues without any outlier are shown as a green connector. A red diamond above a residue indicates a poor fit to the EM map for this residue (all-atom inclusion < 40%).
